# Dishevelled Segment Polarity Protein 3: A Novel Prognosis-related Marker in Pan-driver-gene-negative Lung Adenocarcinoma

**DOI:** 10.7150/jca.87722

**Published:** 2023-09-18

**Authors:** Yongmei Cui, Cuilan Tang, Jinrui Guo, Yu Sun, Zunfu Ke

**Affiliations:** 1 Department of Pathology, The First Affiliated Hospital, Sun Yat-sen University, Guangzhou, Guangdong, 510080, China.; 2 Department of Pathology, The Third Affiliated Hospital, Sun Yat-sen University, Guangzhou, Guangdong, 510080, China.

**Keywords:** “pan-driver-gene-negative” lung adenocarcinoma (PDGN-LUAD), Dishevelled segment polarity protein 3 (DVL3), tissue microarray, stratification analysis, prognosis

## Abstract

**Purpose:** In this study, we aimed to investigate the potential prognostic molecular marker in patients with “pan-driver-gene-negative” lung adenocarcinoma (PDGN-LUAD). LUAD patients who were negative for mutations in EGFR, KRAS, BRAF, HER2, MET, ALK, RET and ROS1 were identified as PDGN-LUAD.

**Methods:** In the screening phase, we profiled the mRNA expression levels in 52 paired PDGN-LUAD tumor tissues and adjacent normal tissues using a genome-wide microarray, and the results revealed that the expression level of dishevelled segment polarity protein 3 (DVL3) was higher in PDGN-LUAD tumor tissues than in normal lung tissues. Then, we enrolled 626 PDGN-LUAD patients from three independent hospital centers and divided them into a training cohort, an internal validation cohort and two external validation cohorts. In the training cohort, tissue microarrays (TMAs) were used to confirm the protein expression level of DVL3. Statistical methods were applied to explore the prognostic role of DVL3.

**Results:** The results indicated that the level of DVL3 could be used to classify patients with PDGN-LUAD in the training cohort into a high-risk group (high expression level of DVL3) and a low-risk group (low expression level of DVL3). In the training cohort, high-risk patients had shorter overall survival (OS) times (hazard ratio [HR] 2.27; 95% CI, 1.57-2.97; p<0.001) than low-risk patients. In the validation phase, the performance of DVL3 as a prognostic marker was successfully validated in the internal and external cohorts.

**Conclusions:** In conclusion, DVL3 is an important prognostic indicator for PDGN-LUAD and may provide new insights into the treatment of PDGN-LUAD.

## Introduction

Lung cancer, a highly invasive and prevalent malignant tumor, is one of the most common diseases that threaten human health. Outcomes for lung cancer patients are often poor, and the 5-year survival rate is less than 17% 1, 2. Lung cancer is divided into two major histopathological subtypes, namely, small cell lung cancer (SCLC) and non-small cell lung cancer (NSCLC). Lung adenocarcinoma (LUAD) is a type of NSCLC. Although the prognosis for NSCLC patients with targetable driver mutations—for example, EGFR mutations and ALK rearrangement—has improved, many patients do not have tumors with mutations in driver genes 3. Patients with pan-driver-gene-negative lung adenocarcinoma (PDGN-LUAD), who are negative for EGFR, KRAS, BRAF, HER2, MET, ALK, RET and ROS1 mutations, still depend on cisplatin and other platinum-based derivatives and immune-checkpoint inhibitors (ICI) as first-line treatments. Combining ICIs with chemotherapy has been shown to improve survival in patients with both squamous and NSCLC, regardless of PD-L1 expression.^4^ When their cancer is resistant to first-line drugs or immune tolerance, PDGN-LUAD patients often have poor prognosis because there are no targeted drugs or other effective therapies. To improve the clinical treatment of PDGN-LUAD, it is important to explore new biomarkers for this subgroup of LUAD patients.

The aberrant reactivation of Wnt signaling pathways has been reported as being involved in the occurrence and prognosis of lung cancer, colon cancer and hepatocellular carcinoma 5-8. Wnt signaling pathways regulate many critical early developmental and postnatal physiological processes, including cell proliferation, differentiation, motility, apoptosis and cellular interactions 9, 10. The WNT/β-catenin pathway is the most thoroughly investigated Wnt signaling pathway, in part due to its important roles in lung development and physiological and pathophysiological processes in the adult lung. Numerous molecules that modulate the Wnt/β-catenin signaling pathway have been proposed as potential targets for lung disease therapies 11-13. The canonical Wnt/β-catenin signaling pathway is initiated when Wnt ligands bind to Frizzled receptors and the coreceptors LRP 5 and LRP 6; the signals are transduced to DVL3, GSK3β, etc. and lead to the inhibition of the phosphorylation of β-catenin in the cytoplasm and its subsequent translocation into the nucleus. Through different Wnt ligands or coreceptors, Wnt may activate β-catenin-independent noncanonical Wnt signaling cascades, including the planar cell polarity (Wnt/PCP) and Wnt/calcium (Wnt/Ca^2+^) pathways. These noncanonical Wnt signaling pathways regulate cell migration, cell polarity and stem cell maintenance.

DVL3 is an essential component in the transduction of the Wnt/β-catenin signaling pathway. At present, there are few reports about the role played by DVL3 in PDGN-LUAD. In this study, we performed a retrospective analysis of 626 patients with PDGN-LUAD. We detected the expression of DVL3 via tissue microarrays (TMAs) and immunoblotting methods and analyzed the association between the expression of DVL3 and the prognosis of PDGN-LUAD patients. The findings of this study shed new light on the diagnosis and treatment of PDGN-LUAD and facilitate the prediction of clinical outcomes for patients with PDGN-LUAD.

## Methods

### Study design

**Figure 1:**
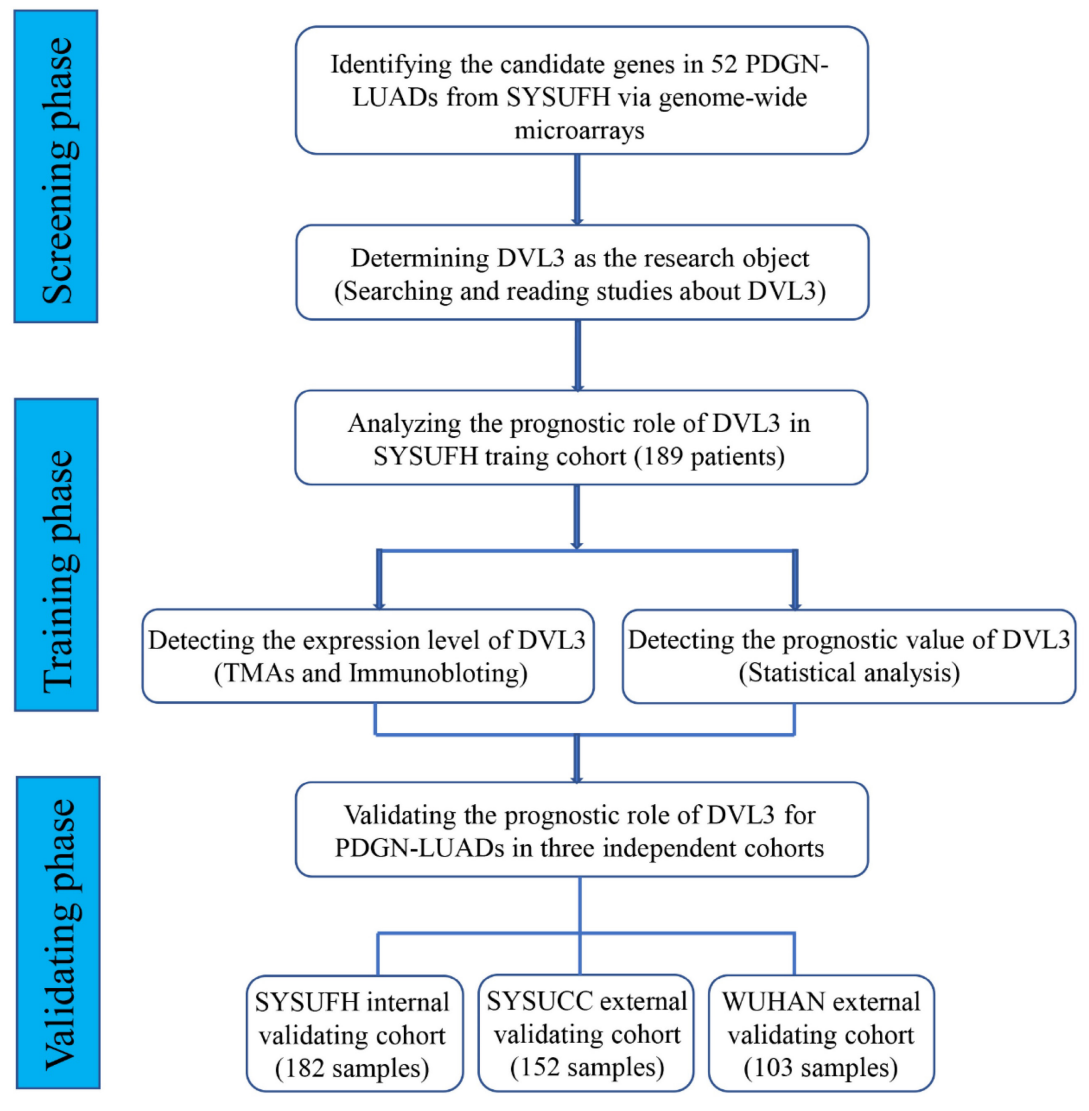
** The study flow chart.** PDGN-LUAD: “pan-driver-gene-negative” lung adenocarcinoma. SYSUFH: The First Affiliated Hospital of Sun Yat-sen University. SYSUCC: Sun Yat-sen University Cancer Center. WUHAN: The Central Hospital of Wuhan.

In this study, we designed three phases to screen, identify and validate prognostic biomarkers for PDGN-LUAD patients. In the screening phase, we profiled the mRNA expression levels in 52 paired PDGN-LUAD tumor and adjacent normal tissue samples using genome-wide microarrays and selected differently expressed gene (DEGs). In the training phase, TMAs and univariate/multivariate Cox regression analysis were used to confirm the prognostic role of the candidate gene. In the validation phase, the performance of the candidate prognostic marker was validated in internal and external cohorts (Figure 1).

### Patients and follow-up

In total, 371 patients at the First Affiliated Hospital, Sun Yat-Sen University (SYSUFH) from April 2006 to July 2015 were enrolled in this study. The patients were randomly divided into training (189 patients) and internal validation (182 patients) cohorts. In addition, 152 patients at the Sun Yat-sen University Cancer Center (SYSUCC) from October 2006 to July 2015 and 103 patients from Wuhan Central Hospital (WUHAN) from August 2006 to July 2015 were enrolled. The entire 626 patients were diagnosed with LUAD by two clinical pathologist, and they were all negative for mutations in the EGFR, KRAS, BRAF, HER2, MET, ALK, RET and ROS1 driver genes. None of the patients underwent any therapy before biopsies were taken. The patients were divided into a training cohort, a SYSUFH internal validation cohort, a SYSUCC external validation cohort and a WUHAN external validation cohort. The control samples were obtained from noncancerous tissue adjacent (>5 cm) to the tumors. The clinical data were collected from the hospital medical records system, including name, sex, age, tumor-node-metastasis (TNM) stage, tumor size, pathological type and degree of differentiation. Most patients, especially patients with stage III/IV LUAD, received multiple treatments, which mainly consisted of chemotherapy with different drugs and radiation therapy with different intensities and cycles; some patients tried Chinese medicine. It would have been extremely difficult to evaluate the influence of the therapeutic schedule on these PDGN-LUAD patients. Hence, in our analyses, we excluded the complicated effect of treatment on the prognosis. Postoperative recurrence and survival time were obtained by outpatient and telephone follow-up. The study was approved by the institutional review board of each hospital center, and was conducted in accordance with the Declaration of Helsinki.

### Genome-wide microarray

Fifty-two pairs of PDGN-LUAD fresh tumor tissue samples and adjacent normal tissue samples were collected from patients at SYSUFH. The genome-wide microarray was performed by Kang Cheng Biological Company. Quantile normalization and subsequent data processing were performed using the GeneSpring GX v12.1 software package (Agilent Technologies). Significantly (p<0.05) DEGs between the 52 pairs of tumor and adjacent normal tissue samples were identified through volcano plots and fold-change filtering. The genome-wide microarray data are available from the National Center for Biotechnology Information (NCBI) Gene Expression Omnibus with the accession number GSE115002.

### Tissue microarrays

To prepare the TMA blocks, a tissue section stained with hematoxylin and eosin (HE) from each patient was prepared; then, representative paired LUAD and adjacent normal tissues were marked on the surface of the donor block according to the coincident diagnoses made by two pathologists. Cylindrical tissue samples with diameters of 2.0 mm were taken from the marked areas of all donor blocks and subsequently placed into one empty recipient microarray block using a precision instrument (Quick-Ray, Korea). Each microarray block contained tissues from 50-56 patients.

### Immunohistochemistry

The formalin-fixed paraffin-embedded (FFPE) samples were cut into 3 μm sections, baked at 65°C for 1-2 hours, deparaffinized with xylene and rehydrated with graded ethanol. Antigens were was repaired by high pressure for 2 min. The sections were incubated in 3% H_2_O_2_ to quench the endogenous peroxidase activity and then incubated with anti-DVL3 (1:100, Abcam, UK) overnight at 4°C. The next day, the sections were incubated with the secondary antibody (ZSGB, China) for 30 min at room temperature, stained with 3,3'-diaminobenzidine (DAB), counterstained with hematoxylin, and finally sealed with neutral balsam. DVL3 was mainly localized in the cytoplasm or cell membrane, and positive expression was determined by the presence of brown granules. The staining signals were quantitatively analyzed with a KS 400 imaging system (Carl Zeiss Vision, Hallbergmoos, Germany). The mean optical density (MOD) of each section was calculated to evaluate the staining intensity of DVL3.

### Immunoblotting

Protein was extracted from fresh tissue samples, and the protein concentration was determined using a BCA assay kit (Kangwei, Beijing, China). The prepared protein samples were separated on a 10% polyacrylamide gel. The proteins were subsequently transferred to a PVDF membrane with a voltage of 100 V for 90 min. The membrane was blocked with 5% nonfat milk at room temperature for 1 h. Then, the anti-DVL3 (1:1000, Abcam, UK) and anti-actin (1:1000, CST, USA) antibodies were added and incubated overnight at 4°C. The next day, the membranes were washed with 1×TBST and then incubated with the secondary antibody (CST, USA) for 1 h at room temperature. Signals were captured using an Enhanced Chemiluminescence Plus kit (Millipore, Billerica, USA).

### Statistical analysis

The measurement data are presented as the means ± standard deviation (SD). *T-*tests and chi-square tests were employed to analyze the differences between groups. Survival analysis was performed using the Kaplan-Meier method, and the survival rates were compared using the log-rank method. Cox regression analysis was used to screen for prognostic factors, and the clinical characteristics were stratified for analysis. X-tile plots were generated with X-tile software version 3.6.1 (Yale University, USA) to determine the optimal cutoff value for DVL3. A time-dependent receiver operating characteristic (ROC) curve was generated to analyze the specificity and sensitivity of DVL3 as a prognostic marker. Statistical tests were performed with R software version 3.0.3 (R Foundation for Statistical Computing). A two-sided p-value<0.05 was considered statistically significant.

## Results

### Patient characteristics

Among the 626 patients with PDGN-LUAD enrolled in this study, 332 were male and 294 were female. In total, 324 patients were younger than 60 years, and 302 patients were older than 60 years. There were 382 patients with lymph node metastasis and 244 patients without lymph node metastasis; 450 patients had TNM stage I/II PDGN-LUAD, and 176 patients had stage III/IV disease. There were 83 patients with well differentiated PDGN-LUAD, 482 patients with moderately differentiated PDGN-LUAD and 61 patients with poorly differentiated PDGN-LUAD. There were 409 patients with tumors smaller than 3 cm in diameter and 217 patients with tumors larger than 3 cm. During follow-up, 464 patients (464/626, 74.3%) experienced progression, and 396 patients (396/626, 63.2%) died. The median overall survival (OS) times were as follows: 56.0 months for patients with stage I disease, 36.0 months for patients with stage II disease, 25.0 months for patients with stage III disease, and 13.0 months for patients with stage IV disease. The clinical characteristics are summarized in Table 1. All patients underwent surgical resection for curative purposes and did not receive any chemotherapy or radiotherapy before surgery.

### DVL3 was overexpressed in patients with PDGN-LUAD in the training cohort

**Figure 2:**
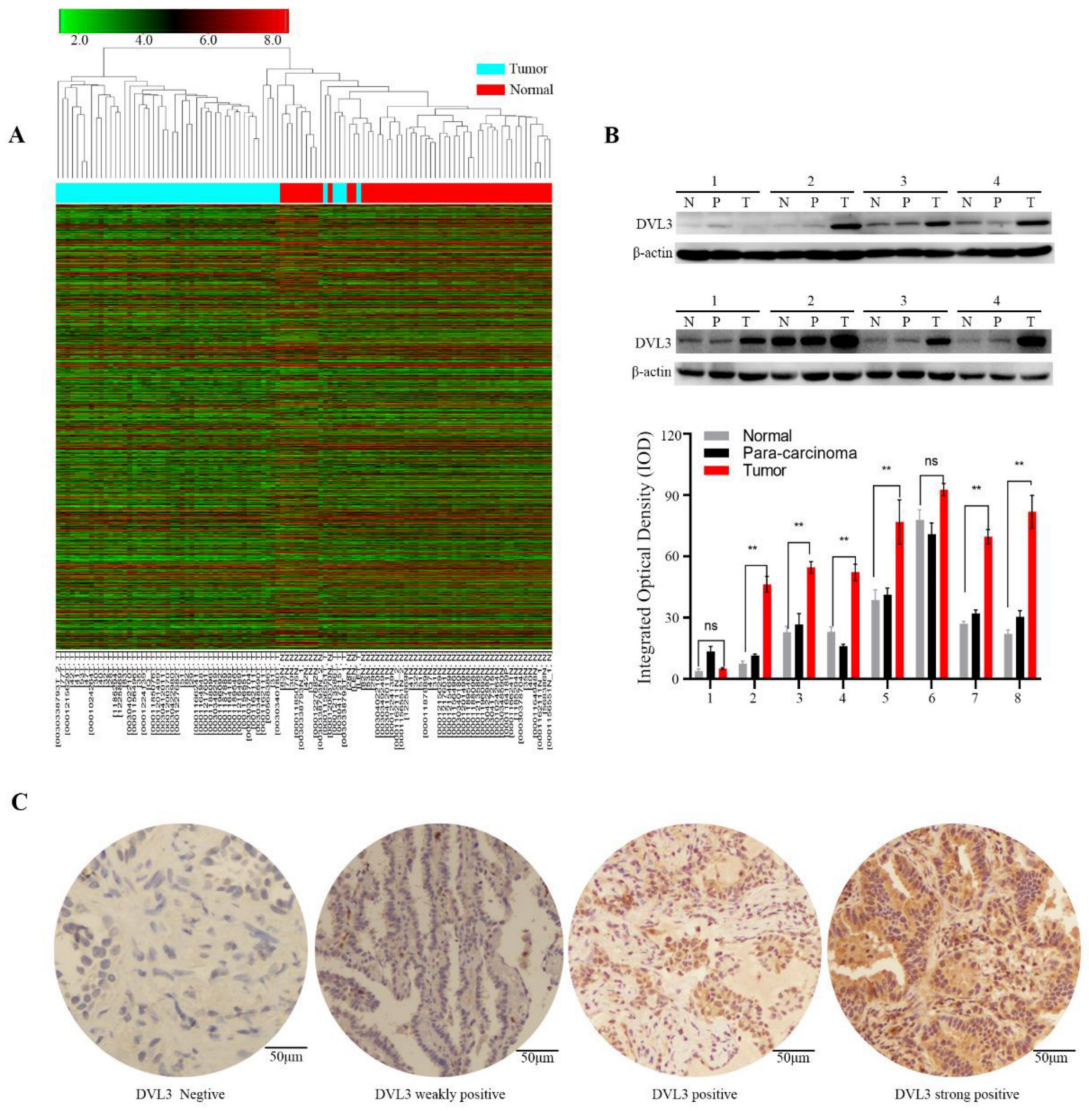
** The expression characteristics of DVL3 in PDGN-LUAD.** (A) Heat map analysis of the genome-wide microarray showing that DVL3 was a candidate gene involved in PDGN-LUAD. (B) The protein expression level of DVL3 was validated by immunoblotting in 8 pairs of representative PDGN-LUAD tissue samples and adjacent normal tissue samples, and the result revealed that DVL3 is overexpressed in tumor tissues compared with normal tissues (p<0.01**). (C) Representative immunohistochemical staining for DVL3. N, normal tissue; P, adjacent normal tissue; T, tumor tissue; *, p<0.05; **, p<0.01; ***, p<0.001.

To identify relevant genes involved in the oncogenesis and development of PDGN-LUAD, we screened the entire human genome and generated a profile that showed genes with different mRNA expression levels between 52 paired PDGN-LUAD and adjacent normal tissue samples via a genome-wide microarray. In total, 960 differently expressed genes (|log2 FC|≥2, p<0.01) were identified, as shown in the heat map in Figure 2A. Among these 960 genes, we chose DVL3 because of its important role in the canonical Wnt/β-catenin signaling pathway and its unknown detailed function in lung cancer. The protein expression level of DVL3 was verified by immunoblotting, and the results indicated that DVL3 was overexpressed in tumor tissues compared with normal tissues (Figure 2B).

**Figure 3:**
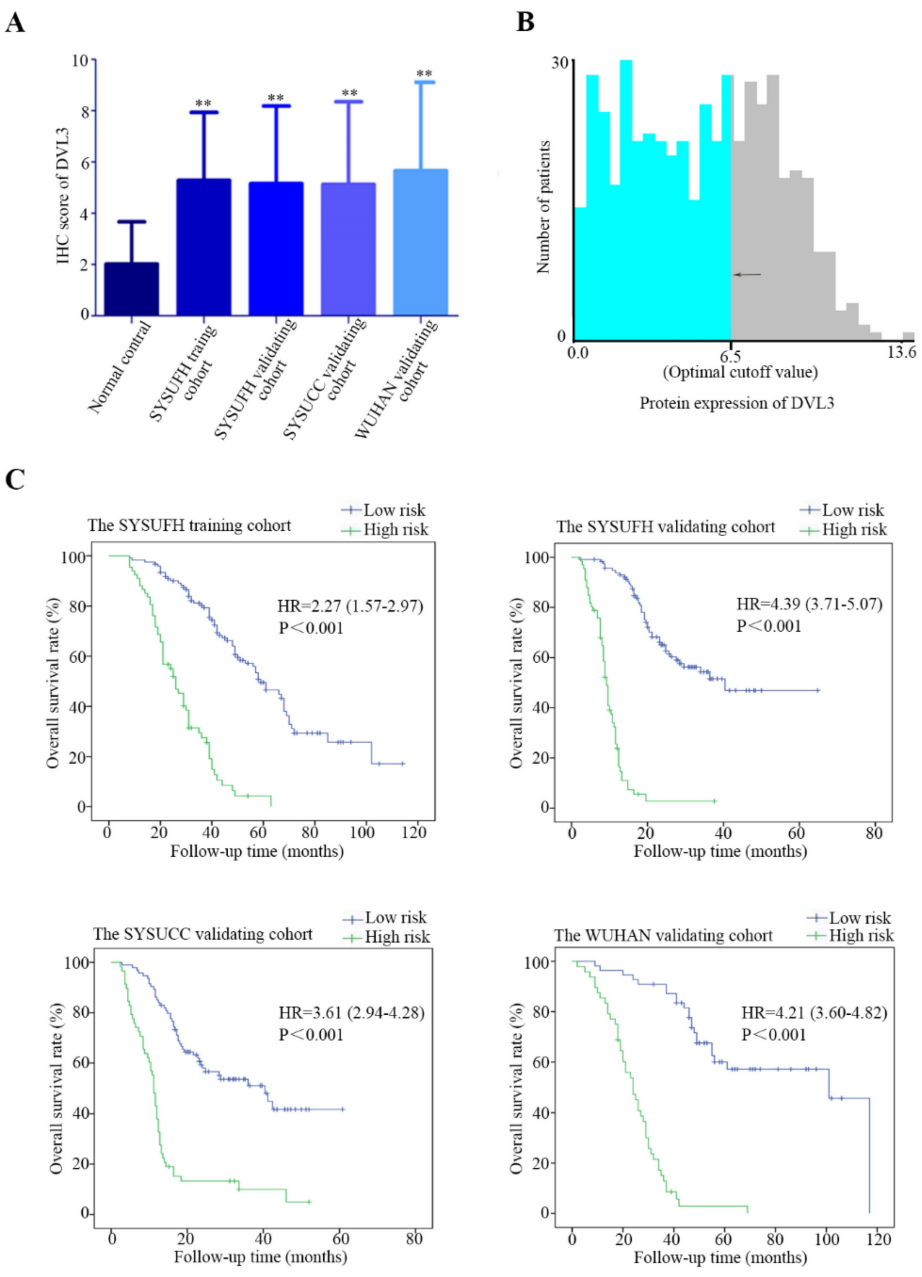
** The optimal cutoff value of DVL3 expression in PDGN-LUAD and Kaplan-Meier estimates of overall survival in the four cohorts based on the expression level of DVL3.** (A) The expression characteristics of DVL3 in PDGN-LUAD patients in the four cohorts. (B) The optimum cutoff value of DVL3 expression signature was determined using the X-tile program in the training set. (C) Application of the DVL3 cutoff value and Kaplan-Meier analyses in the four different cohorts, namely, the SYSUFH training and internal validation cohorts, the SYSUCC external validation cohort, and the WUHAN external validation cohort. HR, hazard ratio; *, p<0.05; **, p<0.01; ***, p<0.001.

We then used immunohistochemistry (IHC) to semi-quantify the protein expression levels of DVL3 in 189 FFPE tissue samples from the training cohort. As shown in Figure 2C, normal lung tissue samples were mostly negative or weakly positive for DVL3, while tumor tissue samples were positive or strongly positive. The DVL3 protein score in the normal control group was 2.02±1.65, and the score in the training cohort was 5.29±2.65 (Figure 3A).

### DVL3 was a prognosis-related biomarker in patients with PDGN-LUAD in the training cohort

The optimum cutoff value for the DVL3 expression level was generated by the X-tile method in the training cohort and was found to be 6.5 (Figure 3B); this cutoff value separated 122 (122/189, 64.6%) patients into the low-risk group and 67 (67/189, 35.4%) patients into the high-risk group (Figure 3C). We performed a t-test and found that the protein expression level of DVL3 in the training cohort was significantly higher than that in the normal control group (P<0.05), as shown in Figure 3A. The distribution of DVL3 risk scores demonstrated that PDGN-LUAD patients with lower risk scores generally had better outcomes than those with higher risk scores (Figure 3C). A survival analysis was performed using the Kaplan-Meier method, and the survival rates were compared with the log-rank test. The OS of the high-risk group in the training cohort was 27.76±1.69 months, and the OS of the low-risk group was 64.56±3.47 months. Then, the high-risk and low-risk groups were analyzed with multivariate Cox regression. As shown in Table 2, the results suggested that DVL3 is an independent prognostic factor for patients with PDGN-LUAD (P<0.001). In summary, DVL3 might be a prognostic biomarker for patients with PDGN-LUAD, and patients with high levels of DVL3 expression (scores ≥ 6.5) have worse prognosis.

### Validation of DVL3 as a prognostic biomarker for patients with PDGN-LUAD

To substantiate the prognostic role of DVL3 in PDGN-LUAD patients, a validation analysis was performed with the SYSUFH internal validation cohort (182 patients) and two external validation cohorts, the SYSUCC external validation cohort (152 patients) and the WUHAN external validation cohort (103 patients). In the internal validation cohort, IHC revealed that the protein expression level of DVL3 was 5.16±3.03. Similar analyses indicated that the DVL3 protein expression levels were 5.13±3.22 and 5.66±3.45 in the SYSUCC and WUHAN external validation cohorts, respectively, as shown in Figure 3A. These data suggested that DVL3 was overexpressed in patients with PDGN-LUAD in the internal validation cohort and the two external validation cohorts when compared to the normal control cohort (P<0.05); this finding was consistent with the results obtained within the training cohort.

According to the cutoff value of DVL3 determined in the training cohort, PDGN-LUAD patients in the internal and external validation cohorts were successfully divided into high-risk and low-risk groups. As shown in Figure 3C, the cutoff value for the DVL3 protein expression level was used to categorize 66 (66/182, 36.3%) patients into the high-risk group and 116 (116/182, 63.7%) patients into the low-risk group in the internal validation cohort. The OS times were significantly different between these high- and low-risk groups (HR=4.39; 95% CI: 3.71-5.07; p<0.001; Figure 3C). Patients in the two external validation cohorts were classified into high-risk and low-risk groups according to the same DVL3 protein expression level cutoff value, and the 94 (94/152, 61.8%) patients with low-risk scores had better OS times than the 58 (58/152, 38.2%) patients with high-risk scores in the SYSUCC external validation cohort (HR=3.61; 95% CI: 2.94-4.28; p<0.001; Figure 3C). In the WUHAN external validation cohort, patients in the low-risk group had better OS times than the patients in the high-risk group (HR=4.21; 95% CI: 3.60-4.82; p<0.001; Figure 3C). When the entire 626 enrolled patients were considered, 387 patients (387/626, 61.8%) were classified as belonging in the low-risk group, and 239 patients (239/626, 38.2%) were classified as belonging in the high-risk group, based on their DVL3 expression scores (HR=2.84; 95% CI: 2.02-3.66; p<0.001; Figure S1). After Cox regression and Kaplan-Meier analyses, we verified that DVL3 remained an independent prognostic factor for OS in the training and validation cohorts (Table 2, Figure 3).

### Stratified analysis

**Figure 4:**
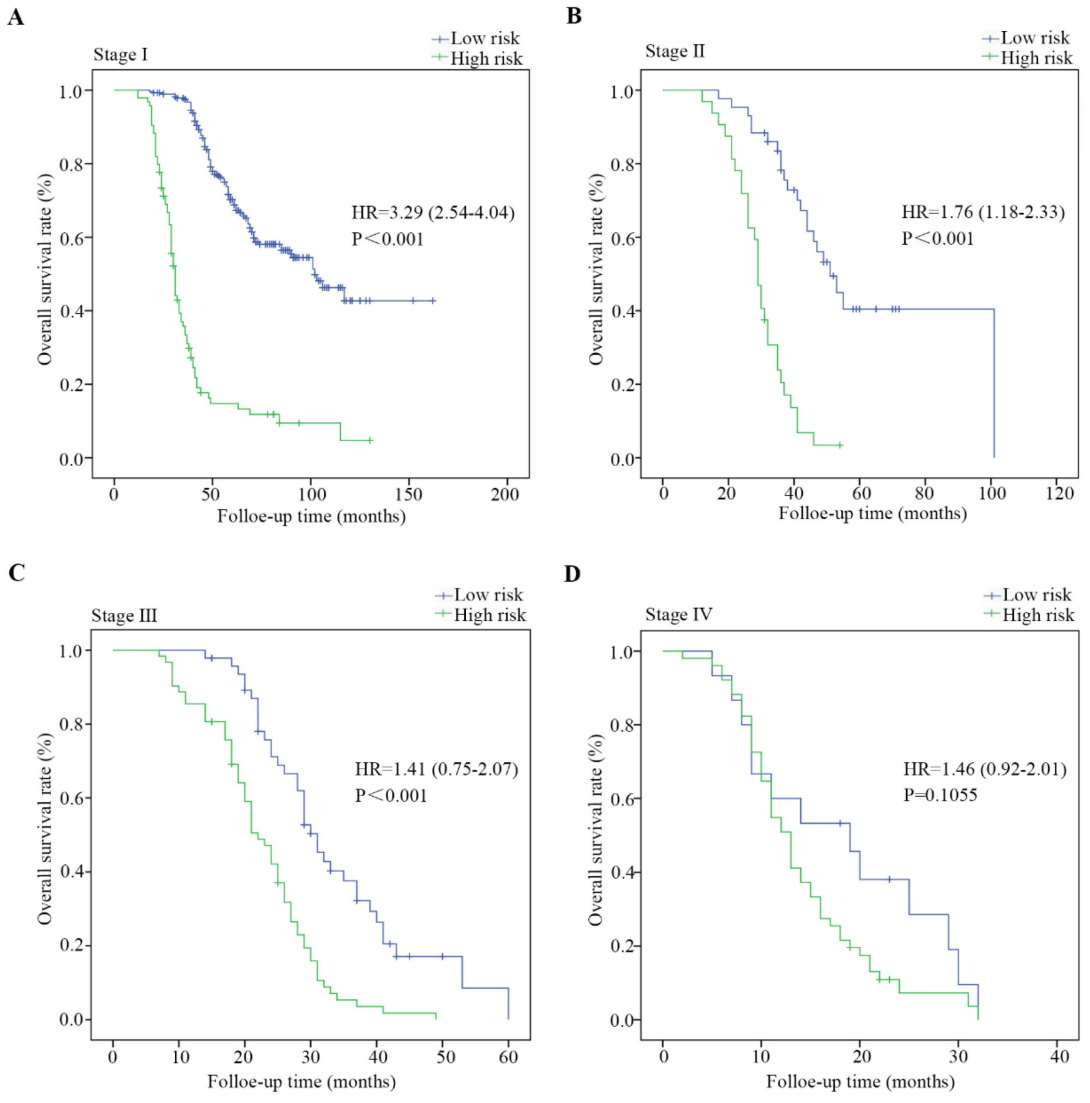
** Kaplan-Meier estimates of overall survival based on the expression of DVL3 in patients with PDGN-LUAD at different TNM stages.** (A) Patients with stage I PDGN-LUAD, (B) patients with stage Ⅱ PDGN-LUAD, (C) patients with stage III PDGN-LUAD, and (D) patients with stage IV PDGN-LUAD. HR, hazard ratio.

The entire cohort of 626 patients was stratified according to clinical characteristics, including age, sex, the degree of differentiation, the presence of lymph node metastasis, tumor size and TNM stage. The survival analysis was performed via the Kaplan-Meier method, and the survival rates were compared with a log-rank test. As shown in Figure 4 and Figure S2, PDGN-LUAD patients within the same stratum of a given clinical characteristic could be divided into high-risk and low-risk subgroups based on the optimal cutoff score of DVL3, and those subgroups had different prognoses. The distinguishing ability was weak for patients with stage IV. This difference may be primarily due to the multimodal treatments for patients with stage IV LUAD which may affect follow-up results. All the findings confirmed that DVL3 is a statistically and clinically significant prognostic biomarker, and it can separate high-risk patients from low-risk patients within each stratum. Furthermore, in the entire cohort of 626 patients, the protein expression levels of DVL3 were strongly associated with the presence of lymph node metastasis and TNM stage (Table 3, all P<0.001); the expression level of DVL3 was higher in patients with lymph node metastasis than in nonmetastatic patients and in patients with stage III/IV disease than in patients with stage I/II disease.

**Figure 5:**
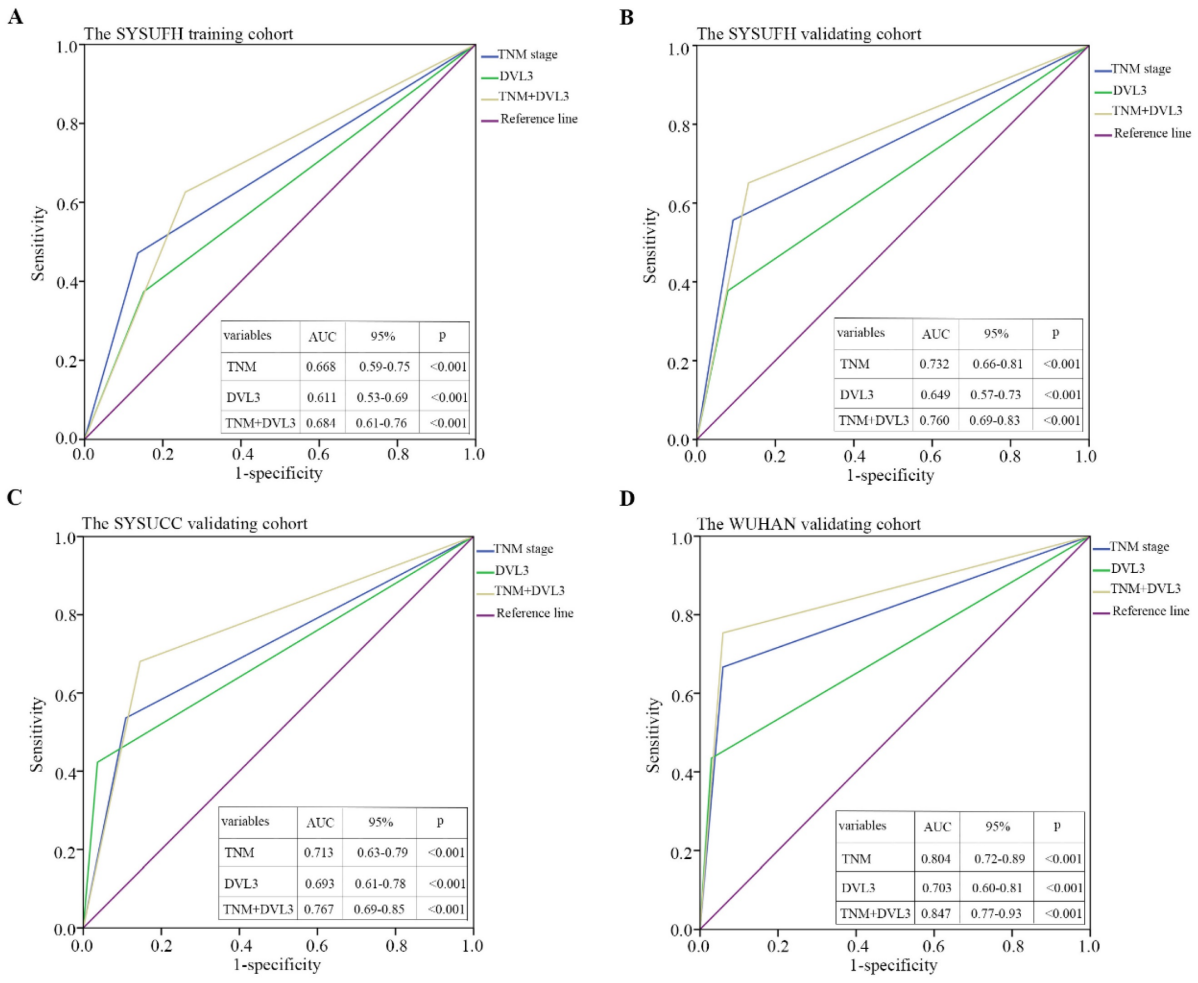
** Time-dependent ROC curves were used to evaluate the sensitivity and specificity of DVL3 for predicting overall survival in PDGN-LUAD patients.** (A) SYSUFH training cohort, (B) SYSUFH internal validation cohort, (C) SYSUCC external validation cohort, and (D) WUHAN external validation cohort. ROC, receiver operator characteristic; AUC, area under the curve; CI, confidence interval.

A time-dependent ROC curve analysis was performed to evaluate the sensitivity and specificity of DVL3 as a predictor of OS in patients with PDGN-LUAD. By analyzing the area under the curve (AUC) for DVL3 in each cohort and the combined group of 626 patients, we found that DVL3 remained a relevant biomarker for OS in patients with PDGN-LUAD; the results were shown in Figure 5 and Figure S3.

## Discussion

Many factors affect the prognosis of LUAD, including the biological characteristics of the tumors, the presence of targetable driver-gene mutations, the immune function of tumor patients and the application of clinical therapeutics. In-depth prognostic analysis is a fundamental prerequisite for the identification of a biological marker that can accurately predict the prognosis of patients with LUAD, especially those with PDGN-LUAD. Previous studies have shown that low expression levels of LKB1 and high expression levels of VEGFR2 may indicate a poor prognosis in patients with LUAD 14, 15, and the loss of expression of p16 and Smad4 in patients with LUAD is related to malignant biological behavior of the tumor and poor prognosis 16. Other studies have shown that CCR4 and PMEPA1 are independent risk factors for the survival of patients with LUAD 17, 18. To enhance the discovery of biomarkers that can effectively predict LUAD, AI-based cancer cytopathology screening represents a valuable complementary approach 19. The discovery of these markers provided a reliable basis for the prognostic evaluation of patients with LUAD.

In the present study, we investigated new prognostic biomarker candidates for patients with PDGN-LUAD. In the screening phase, we profiled mRNA expression levels in 52 pairs of PDGN-LUAD tumor tissue samples and adjacent normal tissue samples using a genome-wide microarray. The results of the Gene Ontology (GO) analysis of the DEGs revealed that most of these candidate genes are involved in the Wnt signaling pathways, extracellular matrix organization, cellular adhesion, epidermal development and osteoblast differentiation. The results of the GO cellular component classification analysis showed that the vast majority of DEGs encode membrane proteins and extracellular matrix components. From these DEGs, we identified four candidate molecules for additional investigation, namely, DVL3, SOX9, Wnt2b and β-catenin. These four genes are involved in the canonical Wnt/β-catenin signaling pathway. SOX9, Wnt2b and β-catenin have been reported in many previous studies 20-22. Several studies have shown that DVL3 can act as a predictor of recurrence in prostate adenocarcinoma 23. Thus, a comprehensive analysis of the role of DVL3 in PDGN-LUAD might provide new insights into the carcinogenesis and development of PDGN-LUAD and lead to the discovery of novel prognostic biomarkers or therapeutic targets for PDGN-LUAD.

*DVLs* are homologs of the Drosophila dishevelled gene and encode cytoplasmic proteins, DVLs, which mainly regulate cell biological behaviors via the Wnt signaling pathway 24. DVLs are cytoplasmic phosphoproteins and are highly functionally and evolutionarily conserved 25. At present, three members of the DVL family have been identified, namely, DVL1, DVL2 and DVL3, and their positions on human chromosomes are 1p36, 17p13.1 and 3q27, respectively 26. Zhao Yue, et al. stated that DVL1 and DVL3 are not expressed in normal bronchial epithelium but are upregulated in lung cancer tissues and that DVL1 and DVL3 could affect the invasion capacity of lung cancer cells 27. Several articles have reported that DVL3 plays an important role in transducing and amplifying signals in the canonical Wnt/β-catenin cascade during tumorigenesis, angiogenesis and progression in patients with NSCLC 28, 29. As a member of the noncanonical Wnt/PCP pathway, DVL3 also integrates signals to induce intracellular cytoskeleton rearrangements 30. Moreover, DVL3 regulates IGF receptor-1 (IGF-IR), a promising new therapeutic target in several cancers, by mediating the IGF-RAS signaling pathway 22. Previous studies demonstrated that DVL3 might be a novel therapeutic target in addition to its prognostic value.

Our study showed that the expression level of DVL3 was significantly higher in PDGN-LUAD tumor tissue than in normal lung tissue, and the abnormal expression of DVL3 was strongly associated with the OS of PDGN-LUAD patients, who could be classified into high-risk and low-risk groups based on their tumor protein expression level of DVL3. Patients in the low-risk group had better OS times than the patients in the high-risk group. The results of univariate and multivariate analyses revealed that DVL3 is an independent prognostic factor for patients with PDGN-LUAD. After the stratified analysis, DVL3 remained a relevant prognostic predictor. We also analyzed the sensitivity and specificity of DVL3 as a prognostic indicator by ROC curve analysis. In summary, DVL3 can be used to estimate the prognosis of patients with PDGN-LUAD.

The limitations of our research should be listed. First, we enrolled patients from China, and thus, the generalizability of DVL3 as a relevant prognostic biomarker for PDGN-LUAD patients worldwide needs to be confirmed. Second, the detailed mechanisms underlying the role of DVL3 in PDGN-LUAD are still unknown. Third, although validation cohorts were used to substantiate the stability of DVL3 as a prognostic biomarker, its clinical utility should be tested in prospective cohorts.

## Conclusion

We revealed that DVL3 was upregulated in PDGN-LUAD, and it could be an appropriate prognostic biomarker for PDGN-LUAD patients. Moreover, in addition to its prognostic value, DVL3 may be a novel therapeutic target for PDGN-LUAD and may help clinicians develop individualized treatment programs for patients with PDGN-LUAD.

## Supplementary Material

Supplementary figures.

## Figures and Tables

**Table 1:** Clinical characteristics of the entire 626 patients from four cohorts.

Characteristics	SYSUFH training cohort	SYSUFH internal validation cohort	SYSUCC external validation cohort	WUHAN external validation cohort
	(n=189)	(n=182)	(n=152)	(n=103)
Age (year)				
≥60: <60	80:109	88:94	79:73	55:48
Sex				
Male: Female	102:87	91:91	85:67	54:49
Tumor size (cm)				
≥3: <3	75:114	62:120	42:110	38:65
Differentiation				
poor	14	14	17	16
moderate	157	147	114	64
high	18	21	21	23
Clinical stage				
I/II: III/IV	133:56	136:46	109:43	72:31

**Table 2:** Multivariate Cox regression analysis of overall survival and clinical characteristics.

Characteristics	SYSUFH training cohort (n=189)		SYSUFH internal validation cohort (n=182)		SYSUCC external validation cohort (n=152)		WUHAN external validation cohort (n=103)	
	HR (95% CI)	p	HR (95% CI)	p	HR (95% CI)	p	HR (95% CI)	p
**Sex (Female: Male)**	1.116 (0.766-1.624)	0.568	0.63 (0.412-0.963)	0.063^*^	1.885 (1.215-2.924)	0.005^*^	1.745(1.032-2.951)	0.058
**Age (≥60 years:<60 years)**	1.030(1.012-1.049)	0.001^*^	1.069(1.046-1.093)	< 0.001^***^	1.047(1.022-1.073)	< 0.001^***^	1.022(0.993-1.053)	0.140
**Differentiation (Low/Median:High)**	1.451 (0.668-3.154)	0.347	0.993 (0.467-2.110)	0.985	1.111 (0.539-2.291)	0.775	1.261 (0.645-2.465)	0.499
**Tumor size (≥3cm:<3cm)**	0.665 (0.449-0.986)	0.042^*^	1.191 (0.760-1.865)	0.446	0.873 (0.538-1.418)	0.583	0.954 (0.570-1.597)	0.858
**Lymphaticmetastasis (Yes: No)**	2.208 (1.283-3.801)	0.004^*^	1.900 (1.075-3.357)	0.027^*^	2.246 (1.186-4.253)	0.013^*^	1.397 (0.626-3.114)	0.034^*^
**TNM stage (III/IV: I/II)**	3.749 (2.140-6.567)	< 0.001^***^	4.313(2.359-7.885)	< 0.001^***^	4.647 (2.460-8.780	< 0.001^***^	3.991 (1.712-9.301)	0.001^***^
**DVL3 (high risk: low risk)**	4.158 (2.698-6.409)	< 0.001^***^	7.036 (4.110-12.045)	< 0.001^***^	4.358 (2.734-6.947)	< 0.001^***^	6.581 (3.252-13.317)	< 0.001^***^

**Table 3:** The correlation between DVL3 and clinical features in patients with PDGN-LUAD.

Factors	SYSUFH training cohort			SYSUFH validating cohort			SYSUCC validating cohort			WUHAN validating cohort		
	Cases	Score	P	Cases	score	P	Cases	score	P	Cases	score	P
**Age**												
<60	109	5.07±2.71	0.175	94	4.32±2.90	<0.001^***^	73	4.84±3.21	0.227	48	5.13±3.43	0.143
≧60	80	5.60±2.57		88	6.06±2.92		79	5.44±3.23		55	6.13±3.43	
**Sex**												
Male	102	5.36±2.60	0.709	91	5.44±3.17	0.222	85	4.83±3.18	0.197	54	6.71±2.88	<0.001^***^
Female	87	5.22±2.73		91	4.89±2.88		67	5.52±3.27		49	4.50±3.68	
**Differentiation**												
High	18	4.72±2.57	0.305	21	3.73±3.36	0.020^*^	21	3.90±2.70	0.135	23	4.92±3.66	0.074
moderate	157	5.28±2.72		147	5.24±2.89		114	5.26±3.24		64	5.50±3.40	
poor	14	6.17±1.69		14	6.55±3.36		17	5.80±3.53		16	7.38±2.95	
**Lymph node metastasis**												
No	106	4.43±2.66	<0.001^***^	119	4.24±2.64	<0.001^***^	92	4.21±3.10	<0.001^***^	65	4.44±3.11	<0.001^***^
Yes	83	6.48±2.15		63	6.91±2.98		60	6.55±2.90		38	7.75±2.99	
**TNM staging**												
Ⅰ/Ⅱ	133	4.64±2.60	<0.001^***^	136	4.37±2.80	<0.001^***^	109	4.41±3.01	<0.001^***^	72	4.73±3.31	<0.001^***^
Ⅲ/Ⅳ	56	6.84±2.08		46	7.51±2.43		43	6.97±3.06		31	7.82±2.77	

## Abbreviations

LUAD: lung adenocarcinoma
PDGN-LUAD: “pan-driver-gene-negative” lung adenocarcinoma
DVL3: Dishevelled segment polarity protein 3
