# Supplementary material for: Dishevelled Segment Polarity Protein 3: A Novel Prognosis-related Marker in Pan-driver-gene-negative Lung Adenocarcinoma
Source: J Cancer. 2023 Sep 18;14(16):3028–38. doi: 10.7150/jca.87722 (PMC10583592; doi:10.7150/jca.87722)
Supplement: Supplementary file 1 — Supplementary figures. [file jcav14p3028s1.pdf]

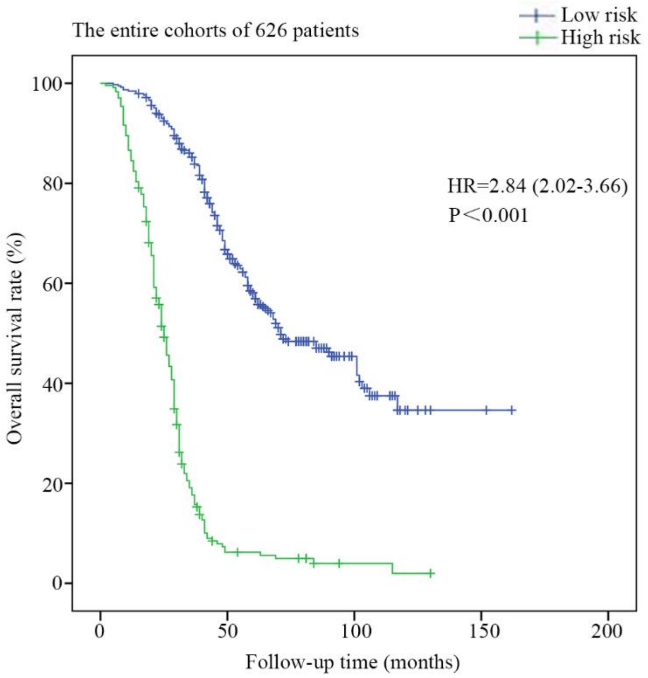

Figure S1 Kaplan-Meier estimates of overall survival in the entire 626 enrolled patients based on the expression level of DVL3. HR, hazard ratio.

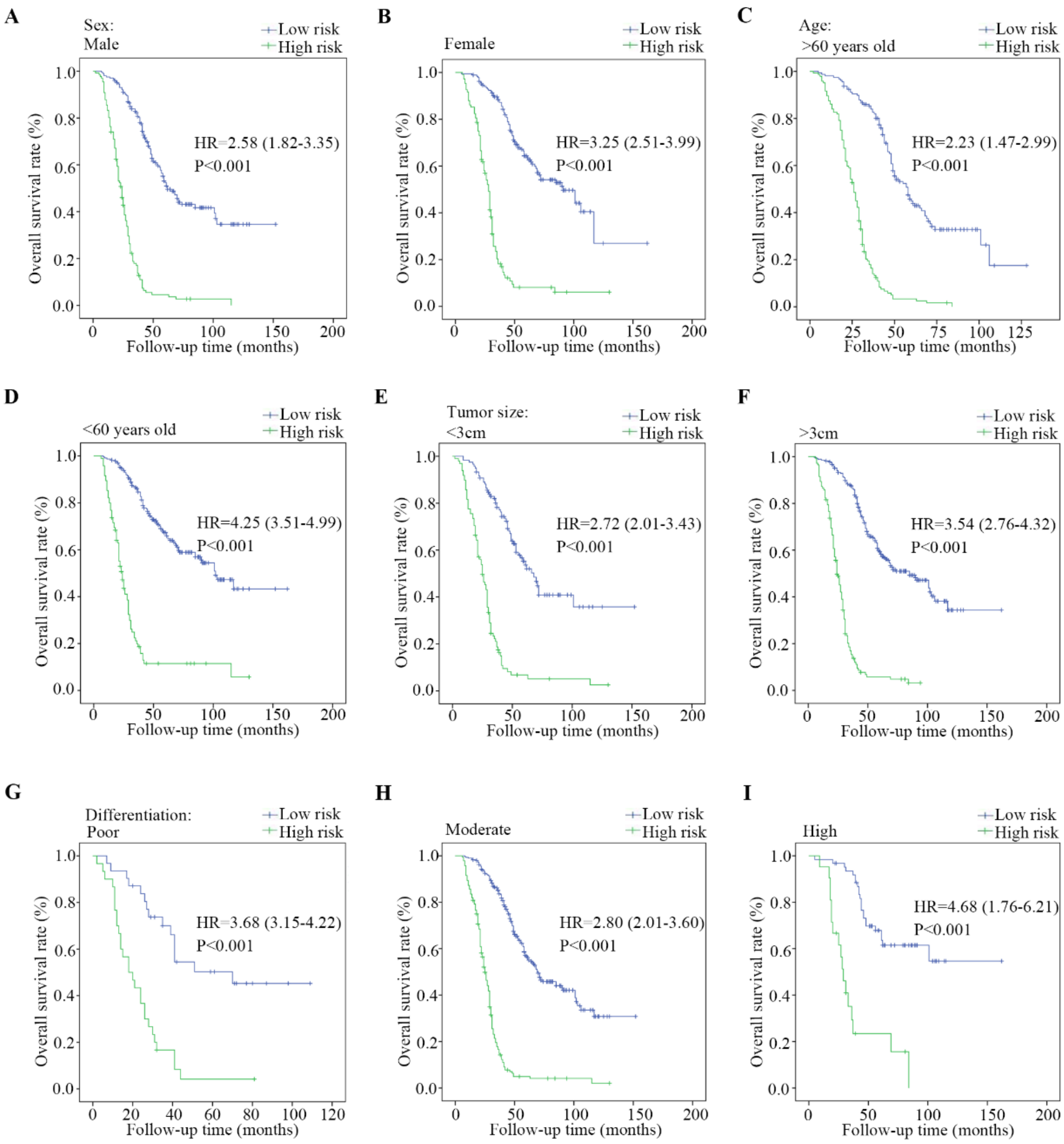

Figure S2 The survival analysis of 626 patients according to clinical characteristics was stratified. (A, B) sex, (C, D) age, (E, F) tumor size, (G, H, I) the degree of differentiation. The survival analysis was performed via the Kaplan-Meier method, and the survival rates were compared with a log-rank test. HR, hazard ratio.

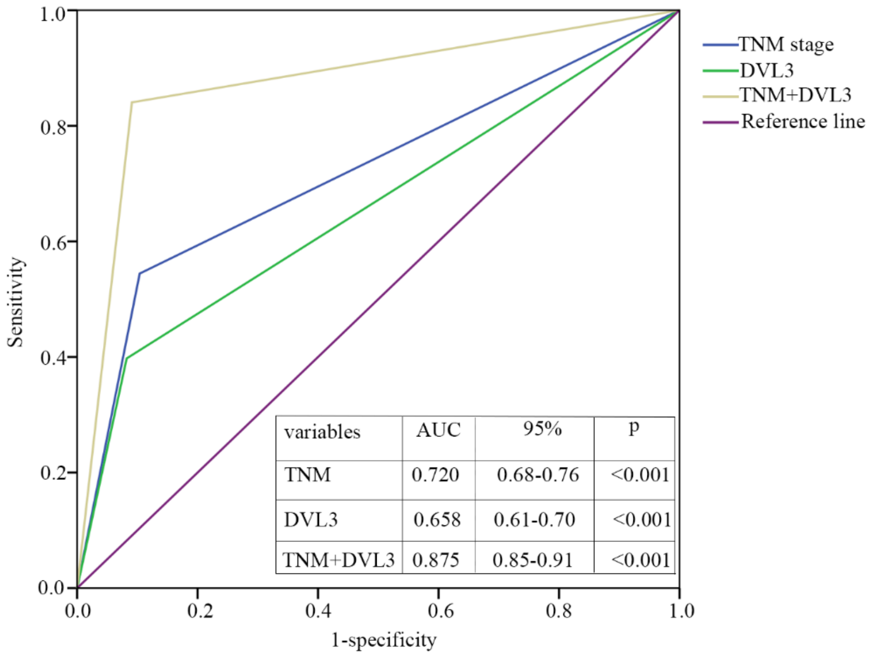

Figure S3 Time-dependent ROC curves were used to evaluate the sensitivity and specificity of DVL3 for predicting overall survival in PDGN-LUAD patients. The entire cohort of 626 patients. ROC, receiver operator characteristic; AUC, area under the curve; CI, confidence interval.
